# Elucidating the interactions between Kinesin-5/BimC and the microtubule: insights from TIRF microscopy and molecular dynamics simulations

**DOI:** 10.1093/bib/bbaf144

**Published:** 2025-04-02

**Authors:** Wenhan Guo, Yuan Gao, Dan Du, Jason E Sanchez, Yupeng Li, Weihong Qiu, Lin Li

**Affiliations:** Department of Physics, University of Texas at El Paso, 500 W University Ave, El Paso, TX 79968, United States; Department of Physics, Oregon State University, 1500 Jefferson Way, Corvallis, OR 97330, United States; Computational Science Program, University of Texas at El Paso, 500 W University Ave, El Paso, TX 79968, United States; Computational Science Program, University of Texas at El Paso, 500 W University Ave, El Paso, TX 79968, United States; Department of Pharmaceutical Sciences, University of Texas at El Paso, 500 W University Ave, El Paso, TX 79968, United States; Border Biomedical Research Center, University of Texas at El Paso, 500 W University Ave, El Paso, TX 79968, United States; Department of Physics, Oregon State University, 1500 Jefferson Way, Corvallis, OR 97330, United States; Department of Physics, University of Texas at El Paso, 500 W University Ave, El Paso, TX 79968, United States; Computational Science Program, University of Texas at El Paso, 500 W University Ave, El Paso, TX 79968, United States; Border Biomedical Research Center, University of Texas at El Paso, 500 W University Ave, El Paso, TX 79968, United States

**Keywords:** kinesin, TIRF microscopy, electrostatics, molecular dynamics, salt bridges

## Abstract

Kinesin-5 s are bipolar motor proteins that contribute to cell division by crosslinking and sliding apart antiparallel microtubules inside the mitotic spindle. However, the mechanism underlying the interactions between kinesin-5 and the microtubule remains poorly understood. In this study, we investigated the binding of BimC, a kinesin-5 motor from *Aspergillus nidulans,* to the microtubule using a combination of total internal reflection fluorescence (TIRF) microscopy and molecular dynamics (MD) simulations. TIRF microscopy experiments revealed that increasing the concentration of KCl in the motility buffer from 0 mM to 150 mM completely abolishes the ability of BimC to bind to the microtubule. Consistent with this experimental finding, MD simulations demonstrated a significant reduction in the strength of electrostatic interactions between BimC and microtubules at 150 mM KCl compared to 0 mM KCl. Furthermore, we identified several salt bridges at the BimC-microtubule interface, with positively charged residues on BimC interacting with negatively charged residues on the tubulin heterodimer. These results provide mechanistic insights into the role of electrostatic interactions in kinesin-5–microtubule binding, advancing our understanding of the molecular underpinnings of kinesin-5 motility.

## Introduction

Kinesins are motor proteins that utilize chemical energy from ATP hydrolysis to generate forces and movement on microtubules [1]. Kinesins are involved in many essential processes, such as intracellular transport and cell division. Based on the sequence similarity, kinesins can be divided into 14 subfamilies (kinesin-1 through kinesin-14) [2]. All kinesins have at least one conserved motor domain, which is responsible for catalyzing ATP hydrolysis and interacting with the microtubule [3, 4]. While most kinesins are homodimers, members of the kinesin-5 all form a dumbbell-shaped homotetramer with pairs of motor domains at both ends of the molecule [5–7]. This unique bipolar structure enables kinesin-5 motors to crosslinking and sliding two different microtubules inside the mitotic spindle. Members of the kinesin-5 subfamily include BimC from *Aspergillus nidulans*, Cin8 from *Saccharomyces cerevisiae*, Cut7 from *Schizosaccharomyces pombe*, KLP61F from *Drosophila*, and HsEg5 from *Homo sapiens* [8–14].

Kinesin-5 proteins have been extensively studied due to their critical roles in mitosis. Many experimental studies have explored their physiological functions, motility and regulatory functions [13, 15–19]. For instance, residue-specific mutations in BimC and HsEg5 have been conducted in multiple studies [10, 20]. Studies have demonstrated that mutations in the Cut7 BimC box region can lead to divergent regulatory behaviors within the kinesin-5 subfamily [20]. It’s worth noting that a single point mutation in the highly conserved region of the BimC box can significantly affect the localization of kinesin-5 on the mitotic spindle [21]. A more recent study highlighted the importance of posttranslational modifications by revealing how C-terminal tail phosphorylation of the Cut7 protein in *Schizosaccharomyces pombe* regulates sliding forces and spindle dynamics during mitotic spindle assembly [22]. Additionally, mutations in the gene encoding BimC in *Aspergillus nidulans* were shown to block nuclear division [10]. While significant progress has been made in characterizing their force generation and spindle pole separation capabilities, the precise mechanisms by which kinesin-5 proteins coordinate their motor activities and modulate their interactions with microtubules are still not fully elucidated. This knowledge gap underscores the complexity of kinesin-5 function and highlights the need for further research to unravel the detailed molecular mechanisms governing their essential roles in cell division.

In recent years, computational methods have been widely used to gain new insights into the mechanisms of different biological system regulations [14, 23–32]. In particular, these methods have been proven to be useful in studying motor proteins [27, 33]. For example, the phylogenetic tree of the kinesin superfamily has been built by adopting parsimony methods derived from sequence alignment techniques [34]. Molecular docking and molecular dynamics approaches have been used to study the inhibition of HsEg5 and other members of in kinesin-5 subfamily by monastrol and related compounds [35]. In a study on the hydrophobic sub-pocket of HsEg5, computational fragment-based drug design was achieved through the MED-SuMo software [36]. We have employed multiscale computational methods to show that electrostatic features on the Eg5 motor domain facilitate the interactions of Eg5 with microtubules, and that salt bridges at the Eg5-tubulin binding interface play a crucial role in stabilizing the complex via an asymmetric binding mechanism [14]. By combining computational approaches with experimental techniques, researchers can gain a deeper understanding of the movement, binding, and other properties of these proteins, offering valuable insights into the role of kinesins in cellular processes and disease mechanisms.

In this work, we focus on investigating the interactions between BimC and the microtubule by combining total internal reflection fluorescence (TIRF) microscopy and molecular dynamics simulation tools including DelPhi [37], DelPhiForce [38, 39], and NAMD [40]. Our TIRF microscopy experiments revealed that while BimC exhibits continuous minus-end-directed motility on the microtubule in the motility buffer with 0 mM KCl, it lacks the ability to bind to the microtubule in the same motility buffer with 150 mM KCl. Consistent with our TIRF microscopy findings, our analyses of electrostatic potential distribution and electrostatic forces showed that the interaction between BimC and the microtubule is significantly stronger at 0 mM KCl than at 150 mM KCl. Notably, molecular dynamics simulations identified several salt bridges at the binding interfaces between the positively charged residues on the motor domain of BimC and the negatively charged residues on the microtubule. Collectively, our findings elucidate the molecular mechanisms governing the kinesin-5/microtubule interaction, offering significant insights into the regulation of kinesins and their potential functions in cellular processes [41–44].

## Methods

### Molecular cloning of BimC(Δ1–70)-GFP

The details of BimC(Δ1–70)-GFP molecular cloning are provided in the Supplementary Information (SI).

### Expression and purification of BimC(Δ1–70)-GFP

The details of BimC(Δ1–70)-GFP expression and purification are provided in the SI.

### Preparation of polarity-marked HiLyte 647 microtubules

We prepared taxol-stabilized HiLyte 647 microtubules with bright plus-ends following the same protocol as described previously [45]. Dim tubulin mix consisting of 17 μM unlabeled tubulin, 0.8 μM HiLyte 647 tubulin and 17 μM biotinylated tubulin was first polymerized in BRB80 buffer (80 mM PIPES, pH 6.8, 1 mM EGTA and 1 mM MgCl_2_), supplemented with 0.5 mM guanosine-50-[(α,β)-methyleno]triphosphate (GMPCPP) at 37°C for 16 h. Following the polymerization, the production mixture was centrifuged at 250 000 g for 7 min at 37°C using a TLA100 rotor (Beckman). The pelleted microtubules were then resuspended in a bright tubulin mix consisting of 7.5 μM unlabeled tubulin, 4 μM HiLyte 647-tubulin, and 15 μM NEM-tubulin in BRB80 with 2 mM GMPCPP. The suspension was incubated at 37°C for 50 min to cap the plus-end of the dim microtubules. To obtain the polarity-marked microtubules, the reaction products were pelleted at 20 000 g for 7 min at 37°C using the TLA100 rotor (Beckman Coulter). The final pellet was resuspended in BRB80 with 40 mM taxol.

### TIRF microscopy

TIRF microscopy experiments were conducted at room temperature using an Axio Observer Z1 microscope (Zeiss). The system was equipped with a 100x 1.46 NA oil-immersion objective and a back-thinned electron multiplier CCD camera (Photometrics). Flow chambers were made by attaching coverslips to microscope glass slides through double-sided tapes. To reduce nonspecific surface absorption of kinesin molecules, all coverslips were functionalized with Biotin-PEG as previously described [46].

### In vitro single-molecule motility assay

In single molecule assays, the flow chamber was treated with 0.5 mg mL^−1^ streptavidin to immobilized polarity-marked HyLite 647 microtubules. BimC(Δ1–70)-GFP was diluted in the motility buffer (BRB15 supplemented with 1 mM ATP, 25 mM taxol, 1.3 mg mL^−1^ casein, and an oxygen scavenger system [47]. The usage of KCl was adjusted according to the need of the experiment). Time-lapse imaging was performed at a frame rate of 1 frame per second. The exposure time and the recording time were set to 100 ms and 5-min accordingly. Kymographs were analyzed in ImageJ (National Institute of Health) to generate the velocity and run-length histograms of BimC(Δ1–70)-GFP, which were then fitted to their respective distribution (Gaussian and exponential).

### Structure preparation for computational study

To investigate how BimC interacts with the microtubule, we employed AlphaFold2 [48, 49] to model its motor domain (residues 71–413). The resulting model yielded pLDDT and pTM values of 87.1 and 0.883, respectively. The pruned atom pairs RMSD (root mean square deviation) between the AlphaFold2 modeled structure and the structure of Human Kinesin-5/Eg5 motor [50] is 1.358 Å, while the RMSD across all pairs is 3.821 Å. With a sequence identity of 52.99%, these results further demonstrate the reliability of the modeled structure (Supplementary Fig. S1). The N-terminal 70 residues outside the motor domain of BimC were not included, because the region was predicted to be highly disordered. Finally, the structure of BimC in complex with a tubulin heterodimer (the BimC-tubulin complex) was constructed using the structure of PDB 6TA4 [50], which represents the motor domain of Eg5 bound to the tubulin in the adenylyl imidodiphosphate state, resolved at a 6.10 Å.

### Molecular dynamics simulations

The BimC-tubulin complex was studied using NAMD [40] with an explicit solvent model. Simulations with a duration of 20 ns were performed on the BimC-tubulin complex both with and without 150 mM KCl (Supplementary Movies 1–2). Based on the RMSD plot (Supplementary Fig. S2), 2000 frames of the simulations (from 10 ns to 20 ns) were selected for further analyses. To ensure the structural insights derived from the 20-ns simulation are robust and representative of the equilibrium state, we extended the simulation to 40 ns and conducted additional analyses to enhance the reliability and confidence of the results. A comparison of the final structures from the 20-ns and 40-ns simulations revealed a RMSD value of 1.049 Å between the 4000th frame (20 ns) and the 8000th frame (40 ns) under the 0 mM KCl condition. Similarly, for the 150 mM KCl condition, the RMSD value between the 4000th and 8000th frames was found to be 1.213 Å. These low RMSD values demonstrate a high degree of structural similarity between the two time points, supporting the stability and consistency of the observed conformations (Fig. S3). The details of the molecular dynamics (MD) simulations are shown in the SI.

### Electrostatic force calculations

To compare the results from MD simulations with experiments and investigate the mechanism of BimC binding with the microtubule, DelPhiForce [38, 39] was employed to compute the electrostatic binding forces between BimC and the tubulin heterodimer. DelPhiForce determines these electrostatic forces by solving the Poisson-Boltzmann equation using the finite difference method:


(1) \begin{equation*} \nabla \bullet \left[\mathrm{\varepsilon} \left(\mathrm{r}\right)\nabla \mathrm{\phi} \left(\mathrm{r}\right)\right]=-4\mathrm{\pi} \mathrm{\rho} \left(\mathrm{r}\right)+\mathrm{\varepsilon} \left(\mathrm{r}\right){\mathrm{\kappa}}^2\left(\mathrm{r}\right)\sinh \left(\frac{{\mathrm{\phi}}\left(\mathrm{r}\right)}{k_BT}\right), \end{equation*}

where $\mathrm{\phi}$(r) is the electrostatic potential, ρ(r) is the charge density, ε(r) is the dielectric permittivity, *κ* is the Debye-Hückel parameter, *k_B_* is the Boltzmann constant, and *T* is the temperature.

For force calculations with and without 150 mM KCl, we used the structures of the BimC-tubulin complex from the 4000^th^ frame (the last frame) of the simulations, as these structures are more reliable after MD simulations [51, 52]. To study the direction and strength of the net forces, DelPhiForce was utilized to calculate the electrostatic forces between the motor domain of BimC and the tubulin heterodimer across varying distances. Using StructureMan [53], the two components were incrementally separated from 10 Å to 40 Å in 2 Å steps. The resulting net forces were visualized using Visual Molecular Dynamics (VMD) [54], as shown in Figs. 2-3.

### Electrostatic potential calculations

To gain deeper insight into the interaction between the motor domain of BimC and the tubulin heterodimer, DelPhi [37] was used to compute the electrostatic potential of the BimC-tubulin complex with 0 mM and 150 mM KCl conditions. DelPhi determines the electrostatic potential of biomolecules by solving the Poisson-Boltzmann equation. In the DelPhi calculation, the protein filling percentage of the grid box was set to 70.0, with a probe radius of 1.4 Å for the molecular structure. The Poisson Boltzmann equation was solved using the dipolar boundary condition. The resulting electrostatic surface potential was visualized using Chimera [55].

## Results and discussion

### Binding of BimC to the microtubule is significantly decreased in high ionic buffers

**Figure 1:**
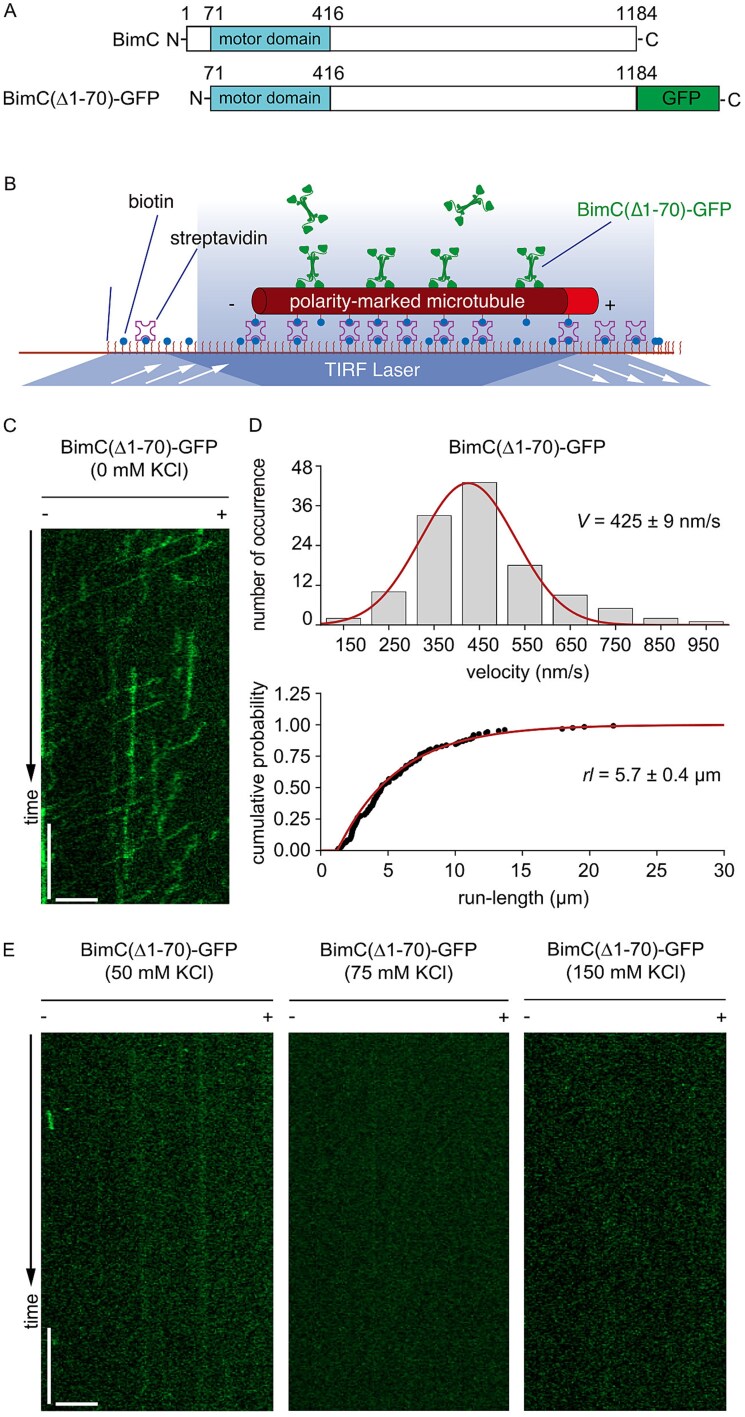
**BimC(Δ1–70)-GFP exhibits drastically different motility with and without 150** mM KCl. (A) Schematic diagrams of the full-length BimC and BimC(Δ1–70)-GFP. (B) A schematic diagram of the TIRF microscopy experiments of BimC(Δ1–70)-GFP on immobilized microtubules. (C) A representative kymograph of BimC(Δ1–70)-GFP at 0 mM KCl concentration. Scale bars: 1 minute (vertical) and 5 μm (horizontal). (D) Top panel: The velocity histogram of BimC(Δ1–70)-GFP at 0 mM KCl (n = 123). The curve represents a Gaussian fit. Bottom panel: The run-length cumulative frequency plot of BimC(Δ1–70)-GFP at 0 mM KCl (n = 123). The curve represents an exponential fit. (E) Representative kymographs of BimC(Δ1–70)-GFP with the same motor input concentration but at three different KCl concentrations (50 mM, 75 mM and 150 mM). Scale bars: 1 minute (vertical) and 5 μm (horizontal).

We set out to examine the binding of BimC to the microtubule under different ionic strengths. To that end, we generated BimC(Δ1–70)-GFP (Fig. 1A), a truncated construct lacking the N-terminal 70 residues outside of the motor domain that is predicted to be highly disordered. We next visualized the motility of BimC(Δ1–70)-GFP using in vitro TIRF microscopy (Fig. 1B). In the motility buffer with 0 mM KCl, BimC(Δ1–70)-GFP bound to microtubules and moved to the minus ends in a processive manner (Fig. 1C; Supplementary Movie 3). Statistical analysis revealed an average velocity of 425 ± 9 nm/s (mean ± s.e., n = 123, Fig. 1D) and an average run-length of 5.7 ± 0.4 μm (mean ± s.e., n = 123, Fig. 1E), indicating a steady interaction between the motor domain of BimC and the microtubule. In contrast, when we repeated the experiments in the motility buffer containing 50 mM KCl, 75 mM KCl or 150 mM KCl, no BimC(Δ1–70)-GFP molecules were observed to land on the microtubule (Fig. 1E; Supplementary Movies 4–6). Collectively, these results suggest that the binding affinity between BimC and the microtubule is negatively impacted by high ionic strength in the motility buffer. It is worth emphasizing that we specifically performed high ionic strength motility experiments at 150 mM KCl, because this concentration corresponds to the typical intracellular ionic strength inside cells [56].

### Increasing ionic strength weakens electrostatic forces between BimC and the microtubule

We next characterized the electrostatic interaction between the motor domain of BimC and the tubulin heterodimer. To do that, we incrementally separated the motor domain of BimC from the tubulin heterodimer at distances ranging from 10 Å to 40 Å in 2 Å steps using StructureMan [53]. At each separation, DelPhiForce [38, 39] was employed to compute the electrostatic forces between BimC and the tubulin heterodimer.

**Figure 2:**
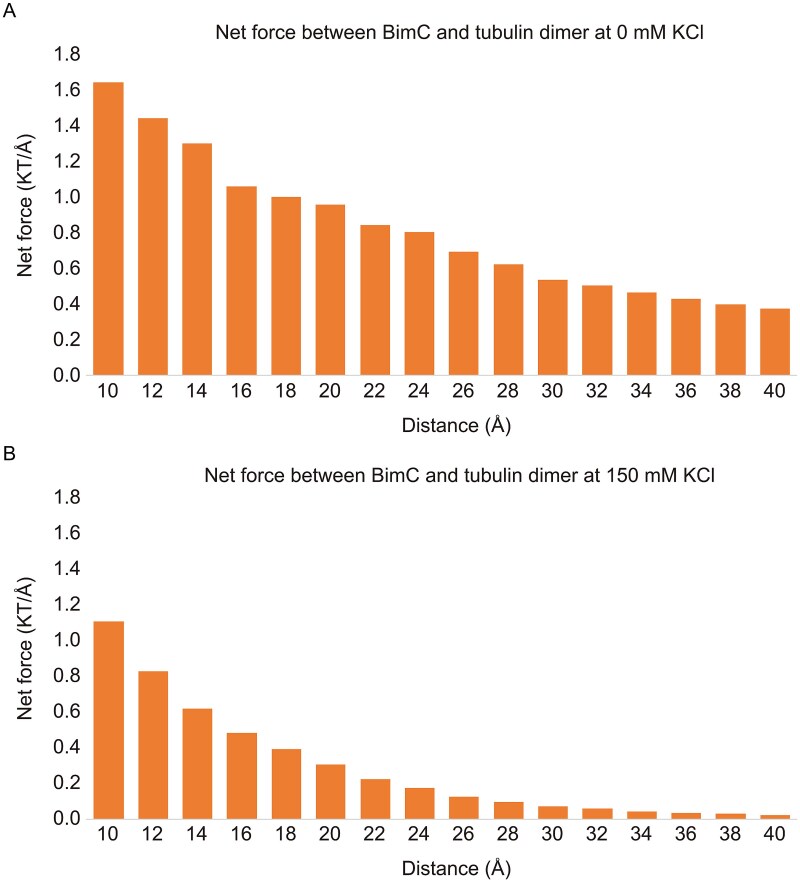
**The net force between BimC motor domain and the tubulin heterodimer at 150** mM KCl is weaker than that at 0 mM KCl. (A) The net forces between BimC and the tubulin heterodimer at 0 mM KCl. (B) The net forces between BimC and the tubulin heterodimer at 150 mM KCl.

The magnitudes of the net binding forces between BimC and the tubulin heterodimer at various separations at 0 mM KCl and 150 mM KCl conditions are shown in Fig. 2. The results indicate that the attractive force decreases as the distance between the motor domain and the tubulin heterodimer increases, which is consistent with Coulomb’s law. The net forces are stronger at 0 mM KCl (which is 1.64 KT/Å) than at 150 mM KCl (which is 1.11 KT/Å), which agrees with our experimental observations that BimC completely lost its binding ability to the microtubule at 150 mM KCl (Fig. 1A; Supplementary Movie 1-2). This finding aligns with the previous study on salt adsorption in molecular complexes [57]. When the KCl concentration surpasses a certain threshold, such as 150 mM, the BimC-tubulin complex can be ‘dissolved’. This is due to the shielding effect on electrostatic interactions between charged components [58, 59].

**Figure 3:**
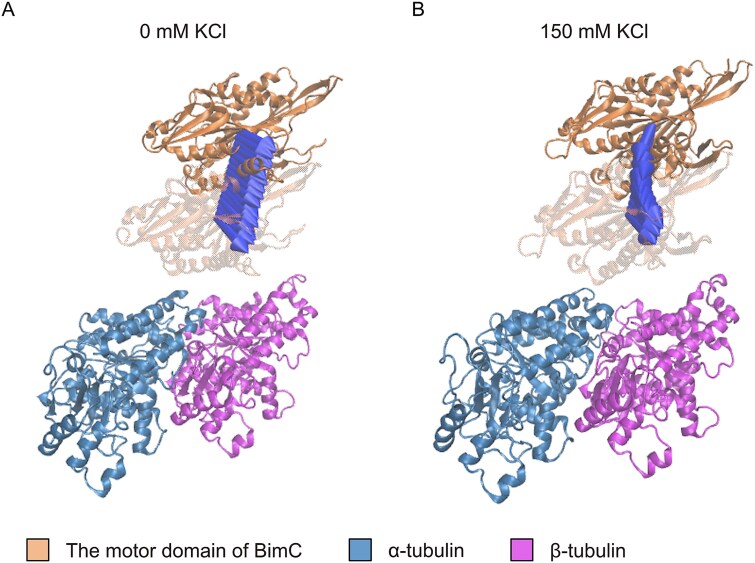
**The tubulin heterodimer exerts a net attractive force on the motor domain of BimC.** (A) The directions of the net electrostatic forces exerted on the motor domain of BimC by the tubulin heterodimer at different distances without 150 mM KCl. (B) The directions of the net electrostatic forces exerted on the motor domain of BimC by the tubulin heterodimer at different distances with 150 mM KCl. These distances vary from 10 Å to 40 Å with a step size of 2 Å. The arrows indicate the directions of the net forces between the motor domain of BimC and the tubulin heterodimer at varying concentrations. To enhance visualization, the arrows are normalized to the same size and thus represent only the force directions, not their magnitudes.

We also used DelPhiForce to determine the directions of the net forces between the motor domain of BimC and the tubulin heterodimer at 0 mM KCl and 150 mM KCl. As shown in Fig. 3, the net forces between BimC motor domain and the tubulin heterodimer (arrows) are predominantly attractive. The strong electrostatic interactions facilitate the binding between these two molecules. Notably, at 0 mM KCl, the electrostatic forces exhibit minimal changes in direction, indicating a more stable and consistent interaction under this condition. In contrast, at 150 mM KCl, the electrostatic forces undergo more pronounced directional shifts, suggesting that higher ionic strength disrupts the electrostatic balance and thus likely weakens the interactions.

### Electrostatic potential reveals BimC-tubulins binding details with and without KCl

**Figure 4:**
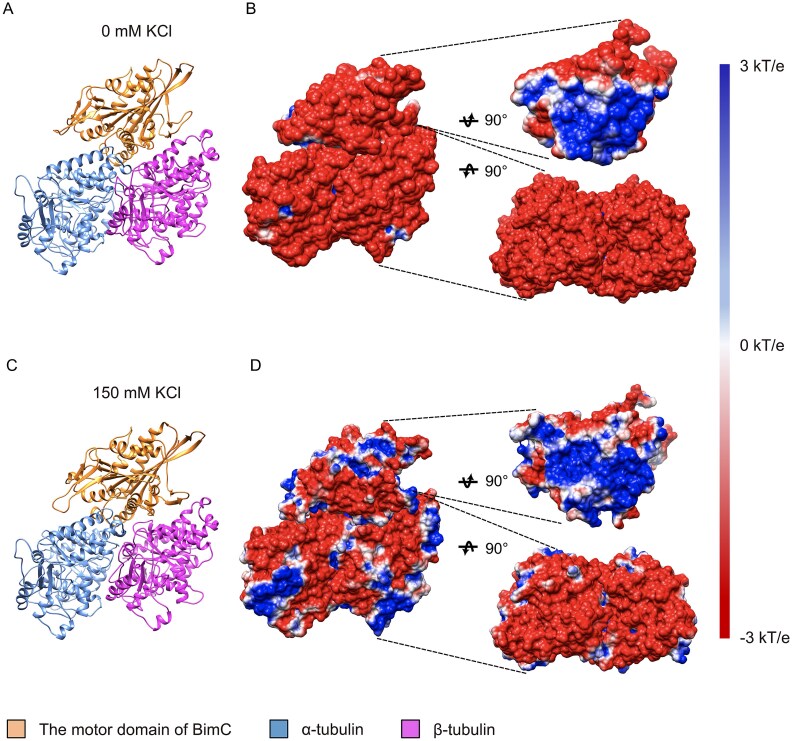
**The BimC-tubulins complex exhibits different electrostatic potential profiles at 0** mM KCl and 150 mM KCl. (A) The structure of the BimC-tubulin complex complexes after the MD simulation without 150 mM KCl. (B) The electrostatic potential profile of the BimC-tubulin complex without KCl. The structure used in the figure is based on the structure in (A). To demonstrate the electrostatic potential profile of the binding interface, the motor domain of BimC and the tubulin heterodimer were rotated 90° in opposite directions on the righthand side. (C) The structure of the BimC-tubulin complex complexes after the MD simulation with 150 mM KCl. (D) The electrostatic potential profile of the BimC-tubulin complex with KCl. The structure used in the figure is based on the structure in (C). To demonstrate the electrostatic potential profile of the binding interface, the motor domain of BimC and the tubulin heterodimer were rotated 90° in opposite directions on the righthand side.

The electrostatic potential provides a map of charged regions on the molecules, allowing us to understand how varying the ionic strength of the solution alters the interaction landscape of the binding complex. This insight is critical because the strength and specificity of molecular interactions often hinge on electrostatic forces, and alteration in these forces can significantly impact the binding affinity, orientation, and overall stability of the complex. By analyzing the electrostatic potential under different conditions, we can better understand the precise molecular mechanisms that drive the binding process, which provide deeper insights into the regulation of kinesins and their roles in cellular functions. Figure 4 shows the structures and electrostatic potential profiles of the BimC-tubulin complexes (with 0 mM and 150 mM KCl). The electrostatic potential profile was computed using DelPhi [37] and mapped onto the molecular surfaces, with the structures rotated at specific angles to highlight the binding interfaces. It is noteworthy that the electrostatic potential profiles were calculated based on the BimC-tubulins complex structures after MD simulations. Positively and negatively charged regions are colored in blue and red, respectively, with a color scale ranging from −3.0 to 3.0 kT/e. The net force between the motor domain of BimC and the tubulin heterodimer is attractive when their interfacial residues possess opposing net charges.

As illustrated in Fig. 4, at 0 mM KCl, the front view of the complex is predominantly negatively charged. With increasing KCl concentration, the electrostatic profile in the side view transitions from a mostly negative distribution at 0 mM KCl to a more intermixed pattern of positive and negative charges at 150 mM KCl. Compared to the structure at 0 mM KCl, the positive and neutral regions in the structure at 150 mM KCl increase in size.

Figure 4 also shows the binding interfaces of each structure rotating 90° of the motor domain of BimC and tubulin protein structures in opposite directions. The motor domain of BimC and the tubulin heterodimer exert attractive forces on each other when they have opposite net charges at the interface. As shown in Fig. 4, the electrostatic potential at the binding interface of BimC is primarily positive at 0 mM KCl. When the concentration of KCl increases, the positively and neutrally charged regions on BimC at the binding interface decrease. As for the tubulin heterodimer, the electrostatic potential at the binding interface of tubulin heterodimer at 0 mM KCl is predominantly negative. When the ionic strength increases, the positively and neutrally charged regions on the tubulin heterodimer at the binding interfaces increase. In conclusion, the electrostatic potential of the BimC-tubulins complex at 0 mM KCl has the large surface area of interacting opposite charges. At 150 mM KCl, the binding interfaces of the BimC-tubulins complex exhibit increased regions of similar charge (either both positively or negatively charged) at the corresponding binding locations, which contributes to the weakening of the electrostatic interaction between BimC motor domain and the tubulin heterodimer. Therefore, the motor domain of BimC and tubulin heterodimer have stronger attractive force at 0 mM KCl than 150 mM KCl. The attractive force may contribute to the stability of the complex, suggesting strong binding affinity. This observation aligns with the electrostatic force analysis results as shown in Fig. 2.

### Salt bridges play a critical role in the interaction between BimC and the microtubule

**Figure 5:**
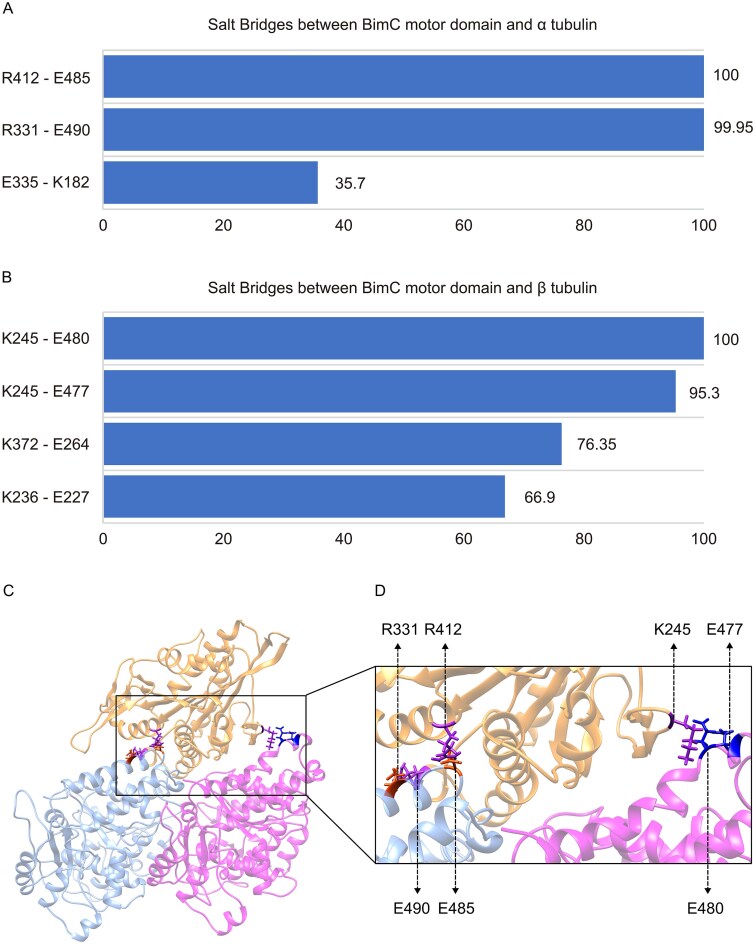
**Salt bridges with high occupancies identified at the binding interface of the BimC-tubulins complex.** (A) Salt bridges with more than 30% occupancies were identified between the motor domain of BimC and α-tubulin. (B) Salt bridges with more than 30% occupancies were identified between the motor domain of BimC and β-tubulin. (C) Positions of salt bridges with more than 90% occupancies between the motor domain of BimC and α/β-tubulin heterodimer. (D) Is the close-up view of (C). The motor domain of BimC, α-tubulin, and β-tubulin are located at the top, left, and right, respectively.

To identify residues involved in the interaction between BimC and the microtubule, we focused on salt bridges with occupancies above 30% at the binding interface of the BimC-tubulins complex. Two salt bridges were found to have occupancies higher than 95% at the binding interface between the motor domain of BimC and α-tubulin at 0 mM KCl (Fig. 5A): the R412-E485 pair formed between R412 in BimC and E485 in α-tubulin, and the R331-E490 pair formed between R331 in BimC and E490 in α-tubulin. Two high-occupancy salt bridges were similarly identified at the binding interface between the motor domain of BimC and β-tubulin at 0 mM KCl (Fig. 5B): the K245-E480 pair formed between K245 in BimC and E480 in β-tubulin, and the K245- E477 pair formed between K245 in BimC and E477 in β-tubulin.

These salt bridges are expected to play a crucial role in stabilizing the binding of BimC to the microtubule, subsequently influencing the motility of BimC along the microtubule. To provide a clearer understanding of the spatial arrangement and significance of these salt bridges, their relative positions are illustrated in Figs. 5C and5D. Visualizing these high-occupancy salt bridges is crucial for comprehensively analyzing the binding mechanism between BimC and the microtubule, since it highlights the key interaction points that are fundamental to this process.

**Figure 6:**
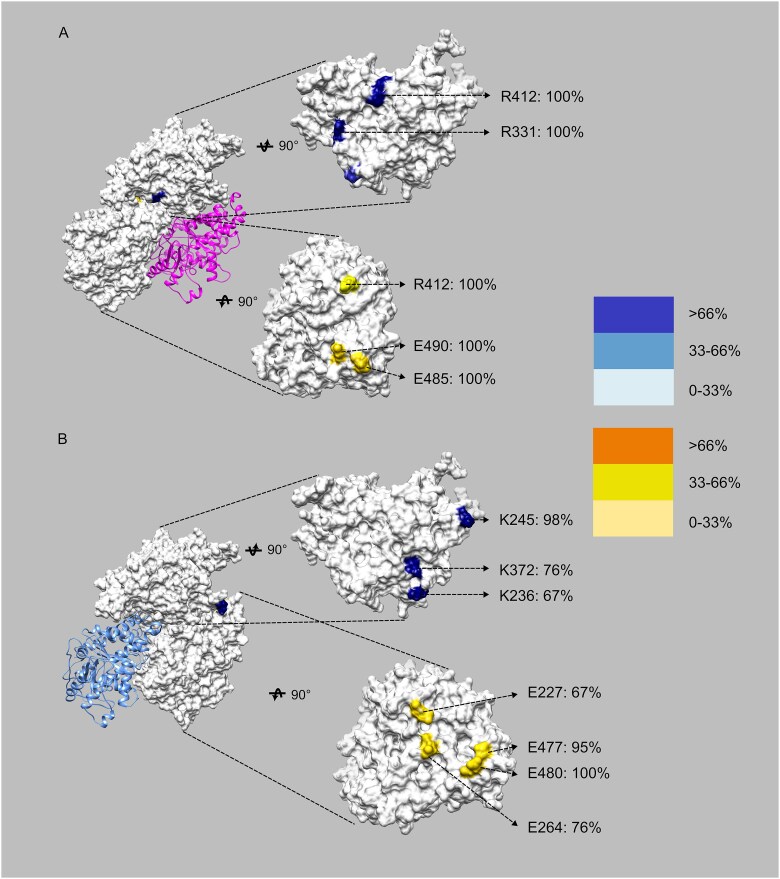
**Salt bridges with high occupancies may contribute significantly to the binding interactions.** (A) Residues involved in the interaction of the motor domain of BimC with α-tubulin. To demonstrate their positions, the motor domain of BimC and the tubulin heterodimer were rotated 90° in opposite directions and shown on the righthand side. (B) Residues involved in the interaction of the motor domain of BimC with β-tubulin. To demonstrate their positions, the motor domain of BimC and the tubulin heterodimer were rotated 90° in opposite directions and shown on the righthand side.

Salt bridges formed by oppositely charged residues are considered as key contributors to maintain the stability and specificity of BimC binding to the microtubule. These electrostatic interactions help to anchor the motor domain of BimC to the microtubule, thereby facilitating its function in cellular processes like mitosis. By analyzing these interactions at 0 mM KCl, we identified the residues that are most consistently involved in forming stabilizing salt bridges. These residues are crucial for pinpointing the specific amino acids that are critical for maintaining the structural integrity of the BimC-tubulins complex. Figure 6 shows the identified residues that form high-occupancy salt bridges at the interface of the BimC-tubulin complex at 0 mM KCl. The residues with occupancies exceeding 66%, which are likely to play a key role in binding interactions, are labeled with their names and occupancy values. R331 and R412 on the motor domain of BimC, along with Q490 and Q485 on α-tubulin, are believed to play a crucial role in stabilizing the interaction between the motor domain of BimC and α-tubulin. K245 and K372 on the motor domain of BimC, and residues Q480 and Q477 on β-tubulin, are critical for the stabilization of BimC binding to β-tubulin. Residues on the motor domain of BimC carry positive charges, whereas those on the microtubule are negatively charged, suggesting that electrostatic interactions play a vital role in the binding between BimC’s motor domain and the microtubule. The residues on BimC identified as key contributors to the BimC-tubulins interaction at 0 mM KCl were mutated to the non-charged residue Ala, leading to a significant decrease in the binding forces (Supplementary Fig. S4). These findings highlight the essential role of these residues in stabilizing the binding interface.

## Conclusions

In summary, we systematically characterized the binding interaction between kinesin-5/BimC and the microtubule under varying ionic strengths by combining TIRF microscopy and computational simulations. Our TIRF microscopy experiments showed that while BimC moves processively toward the microtubule minus ends at 0 mM KCl, its interaction with the microtubule is completely abolished at 150 mM KCl. Guided by these experimental findings, we conducted a series of computational analyses, including MD simulations and electrostatic characterization, to explore the underlying mechanisms. These studies demonstrated that the motor domain of BimC generates a stronger attractive force toward the tubulin heterodimer at 0 mM KCl compared to 150 mM KCl. The computational results align closely with the experimental data, collectively revealing an inverse relationship between ionic strength and binding affinity. Together, these findings provide quantitative insights into how ionic environments regulate motor protein behavior, significantly advancing our understanding of kinesin-5 functions.

Importantly, our study revealed the molecular basis of the BimC-microtubule interaction, by identifying ‘hot spot’ residues that likely contribute to stabilizing the BimC-microtubule complex. Specifically, three residues on BimC (R331, R412, and K245) were found to form salt bridges with negatively charged residues on the tubulins. A deep understanding of the functions of these ‘hot spot’ residues provides valuable insights into the molecular mechanisms underlying kinesin functions and offers a foundation for future efforts to modify or engineer kinesins for research or biotechnological applications [29, 31, 60–63].

While our study provides insights into how ionic strength regulates the interaction between BimC and microtubules, several important questions remain unanswered. For instance, it would be intriguing to investigate whether other kinesin-5 motors, such as Eg5 and Cin8, similarly rely on salt bridges to mediate their interactions with microtubules. Future research should also broaden the scope to include kinesins from other subfamilies, as well as examine the contributions of additional intermolecular forces, such as hydrophobic interactions, to the kinesin-microtubule interaction. Furthermore, targeted mutation experiments focusing on the identified salt bridge residues could help validate their functional significance in vitro and in vivo. Addressing these questions will deepen our understanding of the molecular mechanisms underlying kinesin-microtubule interactions and their broader biological implications.

**Key Points**

- TIRF microscopy experiments showed that increasing KCl concentration from 0 mM to 150 mM markedly decreases the ability of BimC to bind to microtubules.
- Molecular dynamics simulations revealed that electrostatic interactions between BimC and microtubules are significantly weakened at 150 mM KCl compared to 0 mM KCl.
- Our analysis identified a number of salt bridges at the BimC-microtubule binding interface, formed between positively charged residues on the motor domain of BimC and negatively charged residues on tubulins.
- Our findings collectively provide valuable insights into the interaction between kinesin-5 and the microtubule.

## Supplementary Material

BimC_SI_final_bbaf144

Movie1_bbaf144

Movie2_bbaf144

Supplementary_Movie_3_BimC(D1-70)_0mMKCl_bbaf144

Supplementary_Movie_4_BimC(D1-70)_50mMKCl_bbaf144

Supplementary_Movie_5_BimC(D1-70)_75mMKCl_bbaf144

Supplementary_Movie_6_BimC(D1-70)_150mMKCl_bbaf144

## Data Availability

The authors confirm the data of this study is available within the article and supplementary materials.
