# Supplementary material for: Elucidating the interactions between Kinesin-5/BimC and the microtubule: insights from TIRF microscopy and molecular dynamics simulations
Source: Brief Bioinform. 2025 Apr 2;26(2):bbaf144. doi: 10.1093/bib/bbaf144 (PMC11962974; doi:10.1093/bib/bbaf144)
Supplement: BimC_SI_final_bbaf144 [file bimc_si_final_bbaf144.docx]

**Elucidating the Interactions between Kinesin-5/BimC and the Microtubule: Insights from TIRF Microscopy and Molecular Dynamics** **Simulations**

Wenhan Guo^1†^, Yuan Gao^2†^, Dan Du^3^, Jason E Sanchez^3^, Yupeng Li^4,5^, Weihong Qiu^2^*, and Lin Li^1,3,5^*

**^1^** Department of Physics, The University of Texas at El Paso, El Paso, TX

**^2^** Department of Physics, Oregon State University, Corvallis, OR

**^3^**Computational Science Program, The University of Texas at El Paso, El Paso, TX

**^4^** Department of Pharmaceutical Sciences, The University of Texas at El Paso, El Paso, TX

**^5^** Border Biomedical Research Center, The University of Texas at El Paso, El Paso, TX

^†^ **These authors contributed equally to this work.**

*** Correspondence:** Email: [weihong.qiu@oregonstate.edu](mailto:weihong.qiu@oregonstate.edu); [lli5@utep.edu](mailto:lli5@utep.edu).

**Supplementary Methods**

**Molecular Cloning of BimC(Δ1-70)-GFP**

The cDNA of full-length BimC, codon-optimized for protein expression in *Escherichia coli*, was acquired commercially (IDT). The cDNA fragment of BimC lacking the first N-terminal 70 amino acids was integrated into a modified Novagen pET-17b vector containing a 6xHis-tag and a GFP using Gibson Assembly (NEB). The regions corresponding to the 71-1184 amino acids of BimC and the modified vector were separately amplified and then assembled to generate BimC(Δ1-70)-GFP using the KLD Enzyme Mix (NEB). The recombinant construct was verified by DNA sequencing (GENEWIZ).

**Expression and purification of BimC(Δ1-70)-GFP**

The plasmid containing BimC(Δ1-70)-GFP was transformed to and expressed in Novagen Rosetta (DE3) competent cells. Cells were grown at 37 °C in tryptone phosphate medium (TPM) supplemented with 50 μg mL^-1^ ampicillin. Protein expression was induced by 0.1 mM IPTG on ice when OD600 reached 0.8. After incubation for an additional 16 h at 18 °C, cell pellet was harvested at 4,550 g for 30 min using a JS-4.2 rotor (Beckman Coulter). For protein purification, cell pellet was resuspended in the lysis buffer (50 mM sodium phosphate buffer, pH 7.2 with 500 mM NaCl, 1 mM MgCl_2_, 0.5 mM ATP, 10 mM β-mercaptoethanol, 20 mM imidazole, and protease inhibitor cocktail), lysed via sonication at 27,200 g for 30 min using a JA-20 rotor (Beckman Coulter). Soluble protein in the supernatant was puriﬁed by Talon metal affinity resin (Takara Bio) and eluted into the elution buffer (50 mM sodium phosphate buffer, pH 7.2 with 500 mM NaCl, 1 mM MgCl_2_, 0.5 mM ATP, 10 mM β-mercaptoethanol, 250 mM imidazole). Finally, protein was flash-frozen in liquid nitrogen and stored at -80 °C.

**Molecular Dynamics (MD) Simulations**

MD simulations were carried out on Stampede2 at the Texas Advanced Computing Center (<http://www.tacc.utexas.edu>). In the simulation, the minimization was set to 20,000 steps, the temperature was maintained at 300 K, and the pressure was maintained at 1 atm using the Langevin method. The full-system periodic electrostatics coordinates fit the grid size (100, 70, 100). Residues with any atom within 10 Å from the binding interface were treated as interfacial residues. All the interfacial residues were set free while non-interfacial residues were constrained. Simulations were visualized in Visual Molecular Dynamics (VMD) (Supplementary Movies 1-2). To further explore the interactions between the motor domain of BimC and the tubulin heterodimer, salt bridges at the interface at 0 mM KCl were identified and analyzed using the salt bridge extension in VMD. The threshold for salt bridges was set to 4.0 Å. Salt bridges (with occupancy >30%) and interfacial residues (with occupancy >66%) were identified and explored.

**Supplementary Figures**

**
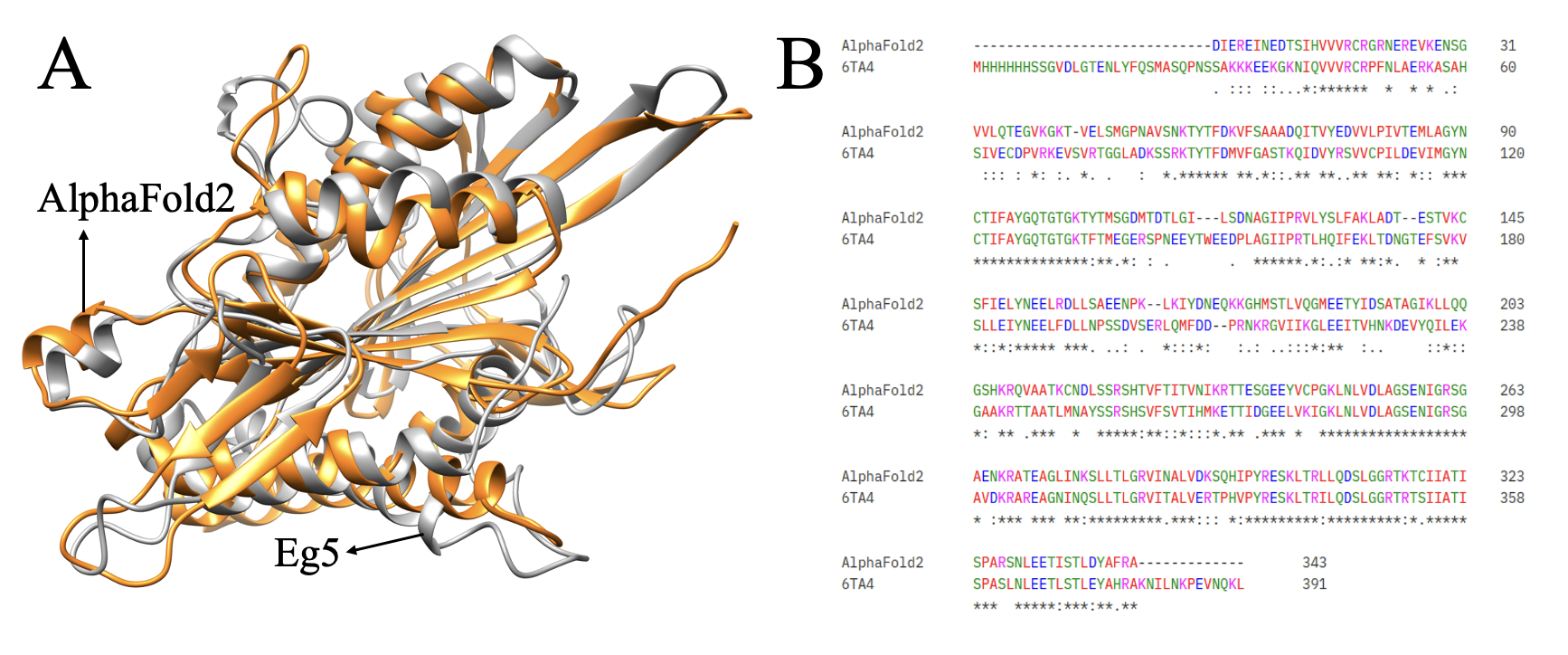
**

**Figure S1:** (A) The side view structure of the AlphaFold2-modeled structure and human kinesin-5/Eg5 motor (PDB entry 6TA4); (B) The sequence alignment of the AlphaFold2-modeled structure and Eg5.


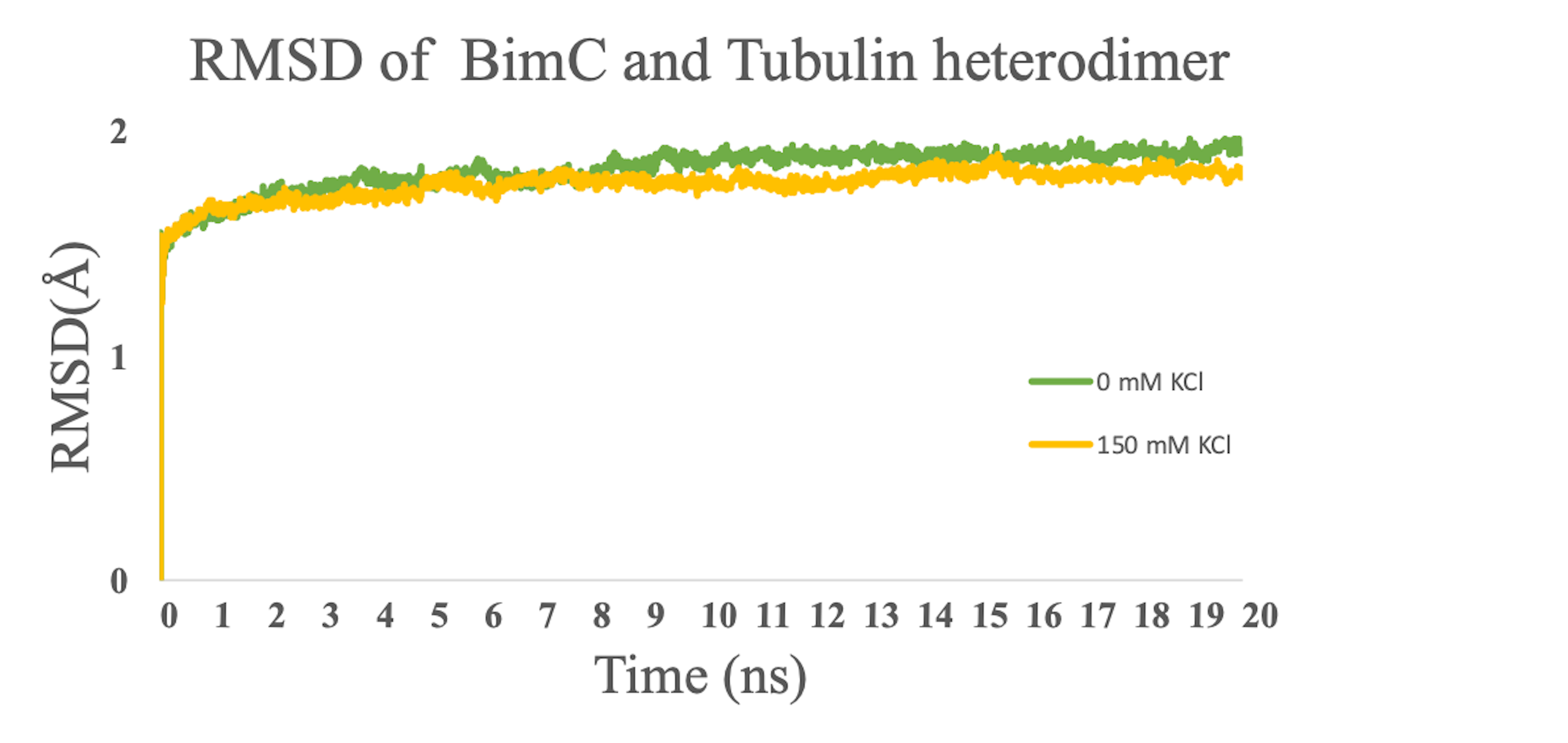


**Figure S2:** **The RMSD of BimC-tubulin heterodimer simulations at the two concentrations of KCl.**


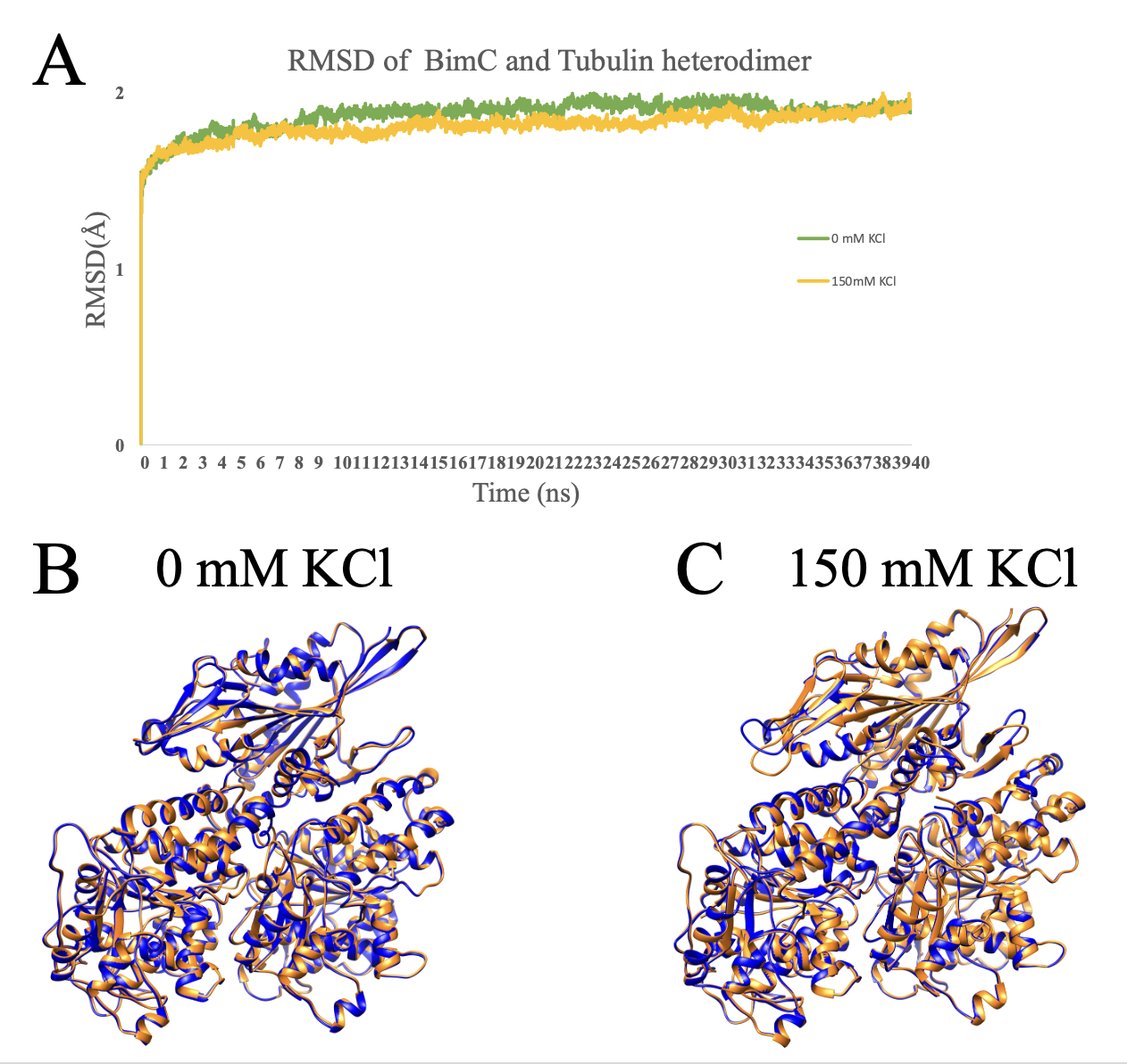


**Figure S3:** **The RMSD and structure comparisons of BimC-tubulin heterodimer extended simulations at two concentrations.** (A) The RMSD of BimC-tubulin heterodimer extended simulations at the 0mM KCl and 150 mM KCl concentrations. (B) The structures of BimC and the tubulin heterodimer from the final frames of the 20 ns and 40 ns simulations at 0 mM KCl. (C) The structures of BimC and the tubulin heterodimer from the final frames of the 20 ns (orange) and 40 ns (blue) simulations at 150 mM KCl.

**Figure S4:** **The net force between the BimC motor domain and the tubulin heterodimer at 0 mM KCl after mutating the BimC residues that were identified as critical contributors to the BimC-tubulin interaction, to the non-charged counterpart Ala.**

Movies:

Movie 1: Simulation of the BimC-tubulin heterodimer (20ns) at 0 mM KCl

Movie 2: Simulation of the BimC-tubulin heterodimer (20ns) at 150 mM KCl

Movie 3: The movie corresponds to the kymograph in Figure 1c. This movie was acquired with BimC(Δ1-70)-GFP at 0mM KCl condition. Under this condition, BimC(Δ1-70)-GFP exhibited minus-end-directed motility on a single polarity-marked HiLyte 647-microtubule. Top: the microtubule channel, and the arrowhead indicates the microtubule plus end; Middle: the BimC(Δ1-70)-GFP channel; Bottom: the overlay of the microtubule and BimC(Δ1-70)-GFP channels.

Movie 4: The movie corresponds to the kymograph on the left panel in Figure 1E. This movie was acquired with BimC(Δ1-70)-GFP at 50mM KCl condition. BimC(Δ1-70)-GFP could not land on the polarity-marked HiLyte 647-microtubule. Top: the microtubule channel; Middle: the BimC(Δ1-70)-GFP channel; Bottom: the overlay of the microtubule and BimC(Δ1-70)-GFP channels.

Movie 5: The movie corresponds to the kymograph on the middle panel in Figure 1E. This movie was acquired with BimC(Δ1-70)-GFP at 75mM KCl condition. BimC(Δ1-70)-GFP could not land on the polarity-marked HiLyte 647-microtubule. Top: the microtubule channel; Middle: the BimC(Δ1-70)-GFP channel; Bottom: the overlay of the microtubule and BimC(Δ1-70)-GFP channels.

Movie 6: The movie corresponds to the kymograph on the right panel in Figure 1E. This movie was acquired with BimC(Δ1-70)-GFP at 150mM KCl condition. BimC(Δ1-70)-GFP could not land on the polarity-marked HiLyte 647-microtubule. Top: the microtubule channel; Middle: the BimC(Δ1-70)-GFP channel; Bottom: the overlay of the microtubule and BimC(Δ1-70)-GFP channels.
